# Disease Control and Prevention in Rare Plants Based on the Dominant Population Selection Method in Opportunistic Social Networks

**DOI:** 10.1155/2022/1489988

**Published:** 2022-01-18

**Authors:** Jia Wu, Fangfang Gou, Xiaoming Tian

**Affiliations:** ^1^School of Computer Science and Engineering, Central South University, Changsha 410083, China; ^2^Hunan Botanical Garden, Changsha 410116, China

## Abstract

The spread of seeds of rare and dangerous plants affects the regeneration, pattern, genetic structure, invasion, and settlement of plant populations. However, seed transmission is a relatively weak research link. The spread of plant seeds is not controlled by the communicator. Rather, this event results from the interaction between the host and the external environment determined by the mother. The way plants transmit and accept seeds is similar to how user nodes accept data transmission requests in social networks. Plants select the characteristics including seed size, maturity time, and gene matching, which are consistent with the size, delay, and keywords of the data received by the user. In this study, we selected rare and endangered Pterospermum heterophyllum as the research object and applied them to a social network. All plants were considered nodes and all seeds as transmitted data. This method avoids the influence of errors in actual sampling and statistical laws. By using historical information to record the reception of seeds, the Infection and Immunity Algorithm (IAIA) in opportunistic social networks was established. This method selects healthy plants through plant social populations and reduces the number of diseased plants. The experimental results show that the IAIA algorithm has a good effect in distinguishing dominant seedlings from seedlings with disease genes and realizes the selection of dominant plants in social networks.

## 1. Introduction

Precious rare plants are plant species that are important in economics, science, culture, and education. In recent years, the number of precious plants has rapidly decreased, resulting in their rarity, with several species becoming extinct. About 20,000–25,000 species of higher plants in the world are on the verge of extinction, accounting for about 10% of the total number of higher plant species [1–4]. The 2015 American Institute of Plant Research reported 761 species of endangered plants, 1,238 threatened species, and 100 extinct species, totaling 1,099 species; this number exceeds 10% of the total number of plant species in the United States. The total number of endangered, threatened, and extinct species in the Hawaiian Islands of the United States has accounted for 49.4% of the total number of plant species in the island. Thus, protecting rare plants has become a common concern among researchers in various industries around the world [5–9].

At present, biologists' research on the protection of cherished plants is mainly focused on the prevention and control of diseases in cherished plants. The existing work is mainly carried out through actual sampling and a large number of statistical laws. However, these research works have the following problems:

1. The work of seedling screening was ignored. Existing work is the study of statistics, that is, the study of plant survival or after survival. This results in a large area of plants being completely random from the seedling stage, and there is no way to screen seedlings at this stage.
2. The effective division of seedlings has not been realized. The work that biologists hope is not only to inhibit the spread of undesirable genes but also to effectively distinguish between dominant seedlings and seedlings with disease genes through prediction and judgment.
3. Seed propagation by wind is the main way for plant populations to reproduce. Through long-term observation of the region, after obtaining statistical predictive indicators such as wind force and wind direction, the potential seedlings of the disease can be separated to avoid the spread of inferior genes in a large area.
4. Plant growth is affected by both genes and the external environment. The existing methods for selecting the best plants through plant marker gene technology are restricted by the plant growth environment. The resulting statistical errors are not conducive to the statistics of dominant plants and the elimination of diseased plants.

Although seed propagation occurs randomly, plants exhibit a propensity for seed selection. The mechanism of plant seed transmission and receipt is similar to how user nodes accept data transmission requests in an opportunistic social network [10]. Plants select the characteristics of seed size, maturity time, and gene matching, which are consistent with the size, delay, and keywords of the data received by the user. Thus, we considered plants to be similar to users and seeds to the data transmitted between users. We can then perform the following comparisons. First, in an opportunistic social network, data transmission is intermittent; the characteristics of seed transmission include mature and intermittently spreading batches. Second, whether the data are received by the user is determined by the data characteristics in the social network. This condition is consistent with plant evolution. Third, in social networks, establishing a trust mechanism and transferring data between the nodes that have cooperated in the past is easily achieved. In plant seed transmission, healthier seeds are more likely to be accepted by other plants, establishing more pollination or breeding cooperation models. Through analysis, we can transform the plant through the pattern of wind-borne seeds into a research method of plant social population in the social network.

Based on the above analysis, this study selected the rare and endangered Pterospermum heterophyllum Dutch plant as the research object and applied it to an opportunistic social network. Among them, all plants are regarded as nodes, and all seeds are regarded as transmission data in the network. In this way, the influence of errors in actual sampling and statistical laws is avoided. Then, this paper proposes an infection and immunity algorithm (IAIA) for plant health seed selection in opportunistic social networks. This method uses historical information to record the receipt of seeds to identify healthy plants. Effective screening is carried out at the plant seed stage to avoid the spread of inferior genes in a large area. In addition, a method for dividing plant ecosystems has been established to effectively classify dominant plants and disease-bearing plants. The IAIA method proposed in this paper effectively reduces the number of diseased plants and realizes the selection of dominant plants in the network.

The contributions of this paper mainly include the following:

1. This study designed a plant ecosystem that describes the social characteristics of wind-borne seeds. The model of wind-borne seeds is transformed into the study of plant populations in opportunistic social networks, and healthy plants are selected through historical records, which realize the effective separation of disease potential seedlings, and enable better selection of dominant plants.
2. This article establishes a method for the classification of plant ecosystems and divides plants into four categories: diseased, susceptible, latent, and immune. It effectively divides plant seedlings and plays an important role in recording the social characteristics of plants.
3. Four groups of impact calculation models are designed. Through the analysis of the four types of plants in the ecosystem, the dominant plants are selected, which effectively improves the activity of healthy immune groups and reduces the transmission efficiency of diseased groups.
4. The experimental results of comparison with classic algorithms show that the IAIA algorithm not only can reduce the impact of disease-borne seed transmission but also improve the overall population receiving healthy seeds. It also has better performance in terms of delivery success rate.

## 2. Related Works

Plant disease detection is the basis for screening healthy plants, and it has always been a hot topic of research by many scholars. Literature [7] proposed an image segmentation algorithm for the automatic detection and classification of plant leaf diseases. It is realized by genetic algorithm, and it also covers the investigation of different disease classification technologies that can be used for plant leaf disease detection. Literature [8] established an algorithm that combines a supervised machine learning algorithm with image processing. The author tested methods such as random forests (RF), support vector machines (SVM), decision trees (DT), and so on. After the experiments, RF had obtained the best classification accuracy after image feature extraction in the tomato disease dataset. Literature [9] proposed a machine learning method combined with a deep neural network (DNN) algorithm to classify the disease degree of tomatoes. The author first used the hybrid principal component analysis (PCA)-whale optimization algorithm to extract a small number of refined key features from the massive features, and then input them into the DNN for classification. This algorithm not only significantly reduces the computational cost of DNN through feature dimensionality reduction but also improves the accuracy of classification.

Literature [10] proposed a plant system. The system described free programmable true-color sensors for real-time recognition and identification of individual weed and crop plants. The application of this type of sensor is suitable for municipal areas and farmland with and without crops to perform the site-specific application of herbicides. Initially, databases with reflection properties of plants, and natural and artificial backgrounds were created. Crop and weed plants should be recognized by the use of mathematical algorithms and decision models based on these data. They include the characteristic color spectrum, as well as the reflectance characteristics of unvegetated areas and areas with organic material.

Literature [11] proposed a robust *PIλDμ* controller method for interval plants. This method is given to show how the presented algorithm can be used to compute all the parameters of a *PIλDμ* controller which stabilizes an interval plant family. Literature [12] proposed a crow search algorithm, which can select the best parameters and exclude irrelevant parameters. The author used it in combination with DNN to improve the accuracy of classification.

Literature [13] presents agro bacterium tumefactions Atu4860 into the gene regulation algorithm; a plant gene regulation algorithm based on agro bacterium tumefactions Atu4860 is realized. Experiments show that the proposed algorithm can effectively improve the quality of plant transgenic, and the implementation process is relatively easy. Literature [14] uses the ensemble–stacking (E-S) method to evaluate and combine versions of machine learning algorithms based on the hyperspectral reflectance of soybeans as a complete or selected input variable to predict soybean yield. Soybean breeders can choose high-yielding soybean genotypes from a large number of genotypes in the early growth stage.

Literature [15] presents a robust plant intelligence-based Adaptive Plant Propagation Algorithm (APPA) which is used to solve the classical ED problem. The application of the proposed method to the 3-generator and 6-generator systems shows the efficiency and robustness of the proposed algorithm. It demonstrates the quality of the solution achieved by the proposed method along with the convergence characteristics of the proposed approach.

In Table 1, we have integrated all the documents and performed a simple comparative analysis. Based on the above research, we integrated plant information with computer science to establish an ecosystem research model.

## 3. System Model Design

In the plant ecosystem, how to guarantee the next generation plants' birth and growth is important, especially in rare plants [16–19]. An illness may cause the extinction of rare plants. The purpose in this work is improving the survival rate of rare plants [20]. We adopt Pterospermum heterophyllum, which is a kind of rare plant in the plant ecosystem to explain the network communication system.

**Figure 1:**
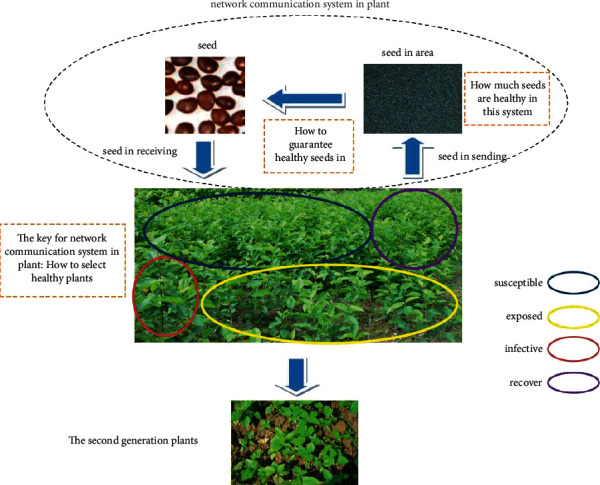
The network communication system in the plant.

Figure 1 shows a Pterospermum heterophyllum network communication system. In this ecosystem, the production of the plant's second generation was released by the plant body and cultured. In the wild ecosystem, the semi-maple seed was spread by the wind. Half-maple will “send” seeds into the air [21, 22]. Seeds are similar to “data packets” in the networks and are “received” by the other half of the “nodes.” When spreading out semi-maple seeds, they can be used as the “source node,” and the half-maple receiving the seeds is the “neighbor node.” During this process, the time of seed release depends on the humidity of the atmosphere, temperature, and oxygen content in the air. If the number of seeds in the air is large, the oxygen content will decrease. The plant will retain a part of the seed, and the seed will be released after the oxygen content has reached the required level. The “carry-transmit-receive” feature of the opportunistic social network was constructed by means of “carrying seed-transporting seed-receiving seed” [23–25].

However, for endangered plants, the next generation of unhealthy seeds exhibits low survival rates. Plants that are self-immune will not only improve their disease resistance but also their reproductive ability. Figure 1 shows that plant systems can be divided into susceptible, exposed, effective, and recover classes. Detecting and filtering “unhealthy nodes,” resisting the seeds of “sick nodes,” and finding the “source nodes” through second-generation plants is an effective method of ensuring population reproduction.

To accomplish the research objective, we need to define several relationships in the plant ecosystem and use them as social network ecosystems [25].

Divide individuals in an ecosystem into four categories:

1. *Class S.* Susceptible, that is, all the uninfected individuals in the ecosystem. If they have effective contact with the virus, they are easily infected and get sick.
2. *Class E*. The exposed class, that is, the entire population of individuals who have been in effective contact with the virus in the ecosystem but have not yet developed the disease. This type of individual is a potential cause.
3. *Class I*. The inefective class, that is, the whole of individuals who have been infected with infectious diseases in the ecosystem. If such individuals have effective contact with individuals of susceptible individuals, it is easy to transmit the disease to the susceptible.
4. *Class R*. Recover class, that is, the entire population of an individual who has contracted an infectious disease and is cured and has immunity in the ecosystem.

Table 2 shows the commonly used symbols and their explanations in this article. To solve the optimization problem (1) quickly, we simplify the hypothesis of the IAIA model:

**Definition 1 .**

The total number of individuals is *N*(*t*)=*S*(*t*)+*E*(*t*)+*I*(*t*)+*R*(*t*), where *S*(*t*) represents the number of susceptible individuals during period *t*; *E*(*t*) represents the number of exposed individuals during period *t*, at which time the disease is contagious; *I*(*t*) indicates the number of individuals infected within the period  *t*, at which time the disease is more contagious; *R*(*t*) indicates the number of individuals who have recovered during the period *t*.

**Definition 2 .**

Initial condition (*S*(0), *E*(0), *I*(0), *R*(0))=(*S*^0^, *E*^0^, *I*^0^, *R*^0^) ∈ *R*_+_^4^, and the parameters  *u*,  *b*,  *ω*_1_,  *ω*_2_, *α*, *β*, *γ* contained in the model, and they are all nonnegative numbers, where 0 ≤ *u*, *b*, *α*, *β*, *γ* ≤  1,  *ω*_1 _ ≥ 1, *ω*_2_ ≥ 1.

**Definition 3 .**

Regardless of the constant input rate of the population (including the individual's birth rate and immigration rate), or the birth rate and mortality of the population, the total number of individuals *N*(*t*)=*N* is constant; *ω*_1_ indicates the exposing period of the disease. After the time *ω*_1_, it may be transformed into an infected one; *ω*_2_ means that the recovered loses immunity after the cycle *ω*_2_, and then transforms into a susceptible one*;β* indicates the effective contact rate of the individual with the disease; *α* indicates the proportion that the individual changes from the exposed state to the infective state; *γ*  indicates the proportion of the infective one into a recovered one; *b* indicates the direct immunization rate of the individual; *u* indicates the proportion of the individual from the exposed state to the recovered state.

**Definition 4 .**

*β*(*E*(*t*)+*I*(*t*))(*t*) represents the proportion of individuals who change from a susceptible one to an exposed one after infection; however, the infected one may be converted into an infective one after the *ω*_1_ period. Therefore, the saturation infection rate *αE*(*t* − *ω*_1_) with time lag is introduced to represent the proportion of exposed individuals those converted into infected individuals after *ω*_1_.

**Definition 5 .**

According to the immunity of the recovery over a while, the rate of introduction of immune loss *γI*(*t*  −  *ω*_2_) indicates the proportion of the recovered who converted to a susceptible state after the immune cycle *ω*_2_.

**Figure 2:**
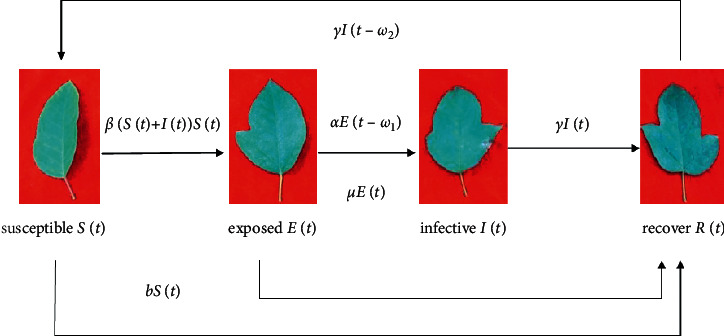
Compartment structure of the IAIA infectious disease model.

Definitions 1to 5 are obtained by simplifying hypotheses 1 to 5 of the IAIA infectious disease model. Hypotheses 1 to 5 of the IAIA infectious disease model are based on the well-known KM hypothesis and consider the characteristics of the IAIA infectious disease with latency. This has been described in detail and will not be repeated here. According to simplified hypotheses 1 to 5, the compartment structure of the IAIA infectious disease model, as shown in Figure 2, is established.

(1) $$\begin{array}{c} \left\{\begin{array}{c} \frac{\text{d}S}{\text{d}t}=-bS\left(t\right)-\beta \left(E\left(t\right)+I\left(t\right)\right)S\left(t\right)+\gamma I\left(t-\omega_{2}\right);\\ \frac{\text{d}E}{\text{d}t}=\beta \left(E\left(t\right)+I\left(t\right)\right)S\left(t\right)-uE\left(t\right)-\alpha E\left(t-\omega_{1}\right);\\ \frac{\text{d}I}{\text{d}t}=\alpha E\left(t-\omega_{1}\right)-\gamma I\left(t\right);\\ \frac{\text{d}R}{\text{d}t}=\gamma I\left(t\right)+uE\left(t\right)+bS\left(t\right)-\gamma I\left(t-\omega_{2}\right). \end{array}\right. \end{array}$$

Suppose the total number of individuals in the ecosystem is 1 unit; Our *S*(*t*), *E*(*t*), *I*(*t*), and *R*(*t*) represent the proportion of individuals in Class S, Class *E*, Class I, and Class *R* in period *t*, respectively. For any individual in the ecosystem, *S*(*t*), *E*(*t*), *I*(*t*), and *R*(*t*) represent the probability that an individual belongs to the Class S, Class *E*, Class I, and Class *R* categories, respectively, or the probability that an individual is in the S state, the *E* state, the I state, and the *R* state, respectively. The S state refers to the uninfected state of an individual, referred to as a susceptible state; the *E* state refers to a state in which an individual has been infected with an infectious disease but has not yet become ill, referred to as an exposed state; the I state refers to an individual who is in the onset state after suffering from an infectious disease, referred to as the onset state; the *R* state refers to the state in which the individual has been infected with the disease and has been cured and has obtained immunity, referred to as the immune state.

Usually, the values of the parameters *u*,  *b*,  *α* ,  *β*,  *γ* are time dependent, but the values of *ω*_1_ and *ω*_2_ are regarded as constants. Since we consider the population in the ecosystem as an individual, we can apply equation (1) to any individual  *i* in the ecosystem and rewrite formula (1) into the following discrete recursive form:

(2) $$\begin{array}{c} \left\{\begin{array}{c} S_{i}\left(t\right)=\left(1-b^{t}\right)S_{i}\left(t-1\right)-\beta^{t}\left(E_{i}\left(t-1\right)+I_{i}\left(t-1\right)S_{i}\left(t-1\right)\right)+\gamma^{t}I_{i}\left(t-1-\omega_{2}\right);\\ E_{i}\left(t\right)=\left(1-u^{t}\right)E_{i}\left(t-1\right)+\beta^{t}\left(E_{i}\left(t-1\right)+I_{i}\left(t-1\right)S_{i}\left(t-1\right)\right)-\alpha^{t}E_{i}\left(t-1-\omega_{1}\right);\\ I_{i}\left(t\right)=\left(1-\gamma^{t}\right)I_{i}\left(t-1\right)+\alpha^{t}E_{i}\left(t-1-\omega_{1}\right);\\ R_{i}\left(t\right)=1-S_{i}\left(t\right)-E_{i}\left(t\right)-I_{i}\left(t\right). \end{array}\right. \end{array}$$

*N*(*t*)=*S*(*t*)+*E*(*t*)+*I*(*t*)+*R*(*t*) is used in formula (2). In the period *t*, the value of each parameter in the formula (2) is as shown in Table 3:

The meaning of Table 3 is: for each period, the value of each parameter is randomly generated within the given value interval [0, 1]. For example, in period *t*, the effective contact rate *β*^*t*^ of the disease is calculated as *β* ^*t*^=Rand(0,  1); Rand(*a*, *b*) indicates that a uniformly distributed random number is generated in the [*a*, *b*] interval. The value of other parameters is similar. The above stochastic method is used to determine the parameters in the IAIA infectious disease kinetic model. Since these parameters are different in different periods, the number of parameter inputs is greatly reduced, and the model can express the actual situation better.

Supposing there are N individuals in an ecosystem, and these individuals are numbered 1,2,…, *N*. Each individual is characterized by *n* features, that is, for individual *i*, its characterization is (*x*_*i*1_, *x*_*i*2_,…, *x*_in_). There is an infectious disease in the ecosystem that will spread between individuals. The epidemic attacks some of the characteristics of the individual, but it is by no means all. The spread of infectious diseases is as follows:

1. An individual in a susceptible state may contract the infectious disease if it is effectively in contact with other individuals who have contracted the infectious disease;
2. The virus in the infected person first enters the exposed state, and the exposed state will continue for a period, which is called the incubation period. A virus that is in an exposed state does not cause an individual to become ill, but if these individuals make effective contact with other individuals, the virus will be transmitted to other individuals;
3. When the body's in vivo virus incubation period is over, the individual begins to become ill, that is, enters the onset state. If the individual in the onset state makes effective contact with other individuals, the virus will be transmitted to other individuals;
4. Individuals in the onset state can be cured with a certain probability; the cured individuals can obtain immunity within a certain period, even if they have effective contact with other individuals who have contracted the infectious disease, they will not transmit the disease. After the individuals' immunization period ends, these individuals will transmit the disease;
5. Individuals in a susceptible or exposed state can gain immunity by receiving a vaccination, but the immunity will disappear after a period.

The physical strength of an individual is determined by the susceptibility of certain characteristics of the individual, the viral latency of certain characteristics, the onset of certain features, and the healing or immunization of certain features. Individuals with strong constitutions can continue to grow, while individuals with weak constitutions stop growing. Mapping the above scenario to the search process for the global optimal solution of the optimization problem formula (1) has the following meanings:

The solution (search) space of the optimization problem formula (1) corresponds to the ecosystem. One individual in the ecosystem corresponds to a tentative solution of the optimization problem formula (1), and the tentative solution set corresponding to *N* individuals is {**X**_1_, **X**_2_,…, **X**_*n*_}.

A feature of the individual *i*(*i*=1,2,…, *N*) corresponds to a variable of the optimization problem tentative solution **X**_*i*_, that is, the feature *j* of the individual *i* corresponds to the variable *x*_*ij*_ of the tentative solution **X**_**i**_, so the feature number of the individual *i* is the same as the number of variables of the tentative solution **X**_**i**_. Therefore, individual *i* and test solution **X**_**i**_ are equivalent concepts. The individual's physical strength is represented by the body mass index *IPI* (individual physique index, *IPI*). The *IPI* index corresponds to the objective function value of the optimization problem formula (1). A good test solution corresponds to an individual with a higher *IPI* value, that is, an individual with a strong constitution, and a poor test solution corresponds to an individual with a lower *IPI* value, that is, an individual with a weak constitution. For the optimization problem formula (1), the calculation method of the individual *i*'s *IPI* index is

(3) $$\begin{array}{c} IPI\left(X_{i}\right)=F_{\max}-F\left(X_{i}\right),\;i=1,2,\ldots ,N. \end{array}$$

In the period  *t*, randomly generate the population of the ecosystem *β*^*t*^, *α*^*t*^, *γ*^*t*^, *b*^*t*^, *u*^*t*^; the IAIA infectious disease model is used to calculate the individual susceptibility probability *S*_*i*_(*t*), exposed probability *E*_ *i*_(*t*), disease probability *I*_*i*_(*t*), and cure probability *R*_*i*_(*t*), which of the four states of the S state, the E state, the I state, and the R state during the period *t* is, respectively, from the biggest one of *S*_*i*_(*t*), *E*_*i*_(*t*), *I*_*i*_(*t*), and *R*_*i*_(*t*).

Since the *β*^*t*^, *α*^*t*^, *γ*^*t*^, *b*^*t*^,  *u*^*t*^ of the ecosystem is time-varying at any time, the susceptibility probability *S*_*i*_(*t*), the exposed probability *E*_*i*_(*t*), the disease probability *I*_*i*_(*t*), and the cure probability *R*_*i*_(*t*) of the individual *i* are all time-varying, so the growth state of the individual *i*  will be randomly converse between the four states S, *E*, I, and *R*.

In the random search process, if the *IPI* index of the individual *i* in the period *t* is higher than the *IPI* index of the period *t* − 1, the individual *i* will continue to grow, which means that the individual *i* is getting closer to the global optimal solution; conversely, if the *IPI* index of individual *i* in period *t* is lower than or equal to the *IPI* index of period *t* − 1, then individual *i* will stop growing, but not shrink, which means that individual *i* stays at the position where period *t* − 1 is not moving. This step-by-step random search strategy makes the algorithm globally convergent.

In period *t*, the state values of *N* individuals of the ecosystem are **X**_1_(*t*), **X**_2_(*t*),…,  **X**_*n*_(*t*), S-S, S-E, S-R, E-E, E-I (*ω*), E-R, I-I, I-R, R-R, and R-S (*ω*) (the design methods of operators are given below. *L* individuals are randomly selected from susceptible, exposed, infective, and recovered individuals, *L*  ≥  1, and these individuals form a susceptible group *C*_*s*_^*t*^={**X**_*i*_1__(*t*), **X**_*i*_2__(*t*),…, **X**_*i*_*i*__(*t*)}, an exposed set *C*_*E*_^*t*^={**X**_*i*_1__(*t*), **X**_*i*_2__(*t*),…, **X**_*i*_*L*__(*t*)}, an infected set *C*_*I*_^*t*^={**X**_*i*_1__(*t*), **X**_*i*_2__(*t*),…, **X**_*i*_*L*__(*t*)}, and a recover set *C*_*R*_^*t*^={**X**_*i*_1__(*t*), **X**_*i*_2__(*t*),…, **X**_*i*_*L*__(*t*)}.

*L* individuals are randomly selected from susceptible, exposed, infective, and recovered individuals, *L*  ≥ 1. The *IPI* index of these individuals is higher than the *IPI* index of the current individual *i*, respectively, forming a cluster of excellent susceptible *C*_PS_^*t*^ = {**X**_*i*_1__(*t*), **X**_*i*_2__(*t*),…, **X**_*i*_*L*__(*t*)}, excellent exposed set *C*_PE_^*t*^ = {**X**_*i*_1__(*t*), **X**_*i*_2__(*t*),…, **X**_*i*_*L*__(*t*)}, excellent patient set *C*_*Pl*_^*t*^ = {**X**_*i*_1__(*t*), **X**_*i*_2__(*t*),…, **X**_*i*_*L*__(*t*)}, and excellent recovery set *C*_PR_^*t*^ = {**X**_*i*_1__(*t*), **X**_*i*_2__(*t*),…, **X**_*i*_*L*__(*t*)}:

(1) *S-S Operator*. Let the difference between the weighted sum of the feature *j* of the *M*_*I*_ susceptible individuals in the set *C*_*s*_^*t*−1^ and its state value and the weighted sum of the features *j* of the *M*_*E*_ susceptible individuals and their state values as the state value of the corresponding feature  *j* of the individual  *i*, which is
  (4) $$\begin{array}{c} \left\{\begin{array}{c} v_{ij}\left(t\right)=\sum_{k=1}^{M_{I}}\alpha_{k}x_{i_{k}j}\left(t-1\right)-\sum_{k=1}^{M_{E}}\beta_{k}x_{i_{k}j}\left(t-1\right),\;\left|C_{S}^{t-1}\right|>0,\\ v_{ij}\left(t\right)=x_{ij}\left(t-1\right),\;{\text{IaIaS}}_{i}\left(t\right)={\text{IaIaS}}_{i}\left(t-1\right),\;\left|C_{S}^{t-1}\right|=0. \end{array}\right. \end{array}$$
**V**_*i*_(*t*) = (*v*_*i*1_(*t*), *v*_*i*2_(*t*),…, *v*_in_(*t*)), **X**_*i*_*k*__(*t* − 1) = (*x*_*i*_*k*_1_(*t* − 1), *x*_*i*_*k*_2_(*t* − 1),……, *x*_*i*_*k*_*n*_(*t* − 1)), *v*_*ij*_(*t*), and *x*_*i*_*k*_*j*_(*t* − 1) are the characteristics of the period *t* and the period *t*  − 1, respectively, the state value of the feature *j* of the individual *i*; ∀*i*_*k*_, *i*_*s*_ ∈ {*i*_1_, *i*_2_,…, *i*_*L*_}, *i*_*k*_ ≠ *i*_*s*_ ≠ *i*; *α*_*k*_, *β*_*k*_ are constants, 0 < *α*_*k*_, *β*_*k*_ < 1, and *α*_*k*_ = Rand(0,1),  *β*_*k*_ = Rand(0,1) during the computing; *M*_*I*_ and *M*_*E*_ are the number of active individuals involved in information exchange, *M*_*I*_ > *M*_*E*_, *M*_*I*_ ≥ 2, *M*_*E*_ ≥ 1, *L* = *M*_*I*_ +  *M*_*E*_; IaIaS_*i*_(*t*) represents the state of the individual *i* at the period *t*, which is one of the four states S, *E*, I, and *R*.
Since the state value of the feature of the individual *i* is calculated without utilizing the characteristics of the individual who is already in other states, the state of the individual *i* does not change; the difference between the weighted sums of the state values of other susceptible individual features is used to calculate the individual *i*'s. The state value of the feature can increase the difference between the individual *i* and other individuals, thereby increasing the degree of difference between the old and new states, resulting in an increase in the activity of the individual *i*.
(2) *S-E Operator.* The characteristic *j* of the *L* individuals in the collection *C*_*E*_^*t*−1^∪*C*_1_^*t*−1^ who are already in an exposed state or have been ill, and their average state values are transmitted to the corresponding features *j* of the susceptible individual  *i* to cause infection with the infectious disease, that is,
  (5) $$\begin{array}{c} \left\{\begin{array}{c} v_{ij}\left(t\right)=\frac{1}{\left|C_{\text{E}}^{t-1}\cup C_{1}^{t-1}\right|}\sum_{k\in C_{\text{E}}^{t-1}\cup C_{1}^{t-1}}x_{kj}\left(t-1\right),\\ \left|C_{\text{E}}^{t-1}\cup C_{1}^{t-1}\right|>0;\\ v_{ij}\left(t\right)=x_{ij}\left(t-1\right),\;{\text{IaIaS}}_{i}\left(t\right)={\text{IaIaS}}_{i}\left(t-1\right),\\ \left|C_{\text{E}}^{t-1}\cup C_{1}^{t-1}\right|=0. \end{array}\right. \end{array}$$
(3) *S-R Operator*. Let the characteristics *j* of the  *L*-cured and immunized individuals in the set *C*_*R*_^*t*−1^ and their average state values be, respectively, transmitted to the corresponding features  *j* of the susceptible individual *i* to obtain immunity, that is,
  (6) $$\begin{array}{c} \left\{\begin{array}{c} v_{ij}\left(t\right)=\frac{1}{\left|C_{R}^{t-1}\right|}\sum_{k\in C_{R}^{t-1}}x_{kj}\left(t-1\right),\left|C_{R}^{t-1}\right|>0;\\ v_{ij}\left(t\right)=x_{ij}\left(t-1\right),\;{\text{IaIaS}}_{i}\left(t\right)={\text{IaIaS}}_{i}\left(t-1\right),\\ \left|C_{R}^{t-1}\right|=0. \end{array}\right. \end{array}$$
(4) *E-I (ω) Operator.* Let the characteristic *j* of the *L* affected individuals in the set  *C*_*I*_^*t*−1^ and its average state value be transmitted to the corresponding feature *j* of the individual *i* who is already in an exposed state, so that the disease occurs, that is,
  (7) $$\begin{array}{c} \left\{\begin{array}{c} v_{ij}\left(t\right)=\frac{1}{\left|C_{I}^{t-1}\right|}\sum_{k\in C_{I}^{t-1}}x_{kj}\left(t-1\right),\;\left|C_{I}^{t-1}\right|>0;\\ v_{ij}\left(t\right)=x_{ij}\left(t-1\right),\;{\text{SEIRS}}_{i}\left(t\right)={\text{SEIRS}}_{i}\left(t-1\right),\\ \left|C_{I}^{t-1}\right|=0. \end{array}\right. \end{array}$$
Because of complex ecological structure, we must consider many cases in ecosystems. Although the state transition of *E*⟶I needs to be delayed by *ω* periods, the state value of the feature of the period *t* individual *i* being attacked by the virus is only related to the state value of the period, *t* − 1. Because when the individual *i* is attacked by the virus, although it occurs only after a certain period of time, its condition is constantly changing during the *ω* period before the onset of the disease.
(5) *E-E Operator*. Let the feature *j* of the *L* individuals in the set *C*_PE_^*t*−1^ and its average state value pass to the corresponding feature *j* of the individual *i* who is already in the exposed state, so that the physical constitution is enhanced, that is,
  (8) $$\begin{array}{c} \left\{\begin{array}{c} v_{ij}\left(t\right)=\frac{1}{\left|C_{\text{PE}}^{t-1}\right|}\sum_{k\in C_{\text{PE}}^{t-1}}x_{kj}\left(t-1\right),\;\left|C_{\text{PE}}^{t-1}\right|>0;\\ v_{ij}\left(t\right)=x_{ij}\left(t-1\right),\;{\text{IaIaS}}_{i}\left(t\right)={\text{IaIaS}}_{i}\left(t-1\right),\\ \left|C_{\text{PE}}^{t-1}\right|=0. \end{array}\right. \end{array}$$
(6) *E-R Operator*. Let the feature *j* of the *L* individuals in the set *C*_PR_^*t*−1^ and its average state value, or the weighted sum of the features *j* and the state values of the *M*_*I*_ individuals in *C*_PR_^*t*−1^ and the characteristics of the other *M*_*E*_ individuals in *C*_PR_^*t*−1^ and their states. The difference between the weighted sums of the values is taken as the state value of the feature  *i* corresponding to the individual *i*, which not only cures and gains immunity but also increases its activity, that is,
  (9) $$\begin{array}{c} \left\{\begin{array}{c} \begin{array}{c} \begin{array}{c} v_{ij}\left(t\right)=\frac{1}{\left|C_{\text{PR}}^{t-1}\right|}\sum_{k\in C_{\text{PR}}^{t-1}}x_{kj}\left(t-1\right),\\ \text{Rand}\left(0,1\right)<0.5,\;\left|C_{PR}^{t-1}\right|>0;\\ \begin{array}{c} v_{ij}\left(t\right)=\sum_{k=1}^{M_{1}}\alpha_{k}x_{i_{k}j}\left(t-1\right)-\sum_{k=1}^{M_{\text{E}}}\beta_{k}x_{i_{k}j}\left(t-1\right)\\ \text{Rand}\left(0,1\right)\ldots 0.5,\;\left|C_{PR}^{t-1}\right|>0; \end{array} \end{array}\\ v_{ij}\left(t\right)=x_{ij}\left(t-1\right),\;{\text{IaIaS}}_{i}\left(t\right)={\text{IaIaS}}_{i}\left(t-1\right),\\ \left|C_{\text{PR}}^{t-1}\right|=0. \end{array} \end{array}\right. \end{array}$$
Equation (9) not only integrates the characteristics of the S-S operator and the E-E operator but also avoids the similarity with the S-R operator.
(7) *I-I Operator*. Let the feature *j* of the *L* individuals in the set *C*_PI_^*t*−1^ and its average state value pass to the corresponding feature *j* of the diseased individual *i* to enhance its constitution, that is,
  (10) $$\begin{array}{c} \left\{\begin{array}{c} v_{ij}\left(t\right)=\frac{1}{\left|C_{\text{PI}}^{t-1}\right|}\sum_{k\in C_{\text{PI}}^{t-1}}x_{kj}\left(t-1\right),\;\left|C_{\text{PI}}^{t-1}\right|>0;\\ v_{ij}\left(t\right)=x_{ij}\left(t-1\right),\;{\text{IaIaS}}_{i}\left(t\right)={\text{IaIaS}}_{i}\left(t-1\right),\\ \left|C_{\text{PI}}^{t-1}\right|=0. \end{array}\right. \end{array}$$
(8) *I-R O perator*. Let the feature *j* of the *L* individuals in the set *C*_*R*_^*t*−1^ and its average state value, or the weighted sum of the features *j* and the state values of the *M*_*I*_ individuals in *C*_*R*_^*t*−1^ and the characteristics of the other *M*_*E*_ individuals in *C*_*R*_^*t*−1^ and their states. The difference between the weighted sums of the values is taken as the state value of the feature  *i* corresponding to the individual *i*, which not only cures and gains immunity but also increases its activity, that is,
  (11) $$\begin{array}{c} \left\{\begin{array}{c} \begin{array}{c} \begin{array}{c} v_{ij}\left(t\right)=\frac{1}{\left|C_{R}^{t-1}\right|}\sum_{k\in C_{R}^{t-1}}x_{kj}\left(t-1\right),\\ \text{Rand}\left(0,1\right)<0.5,\;\left|C_{R}^{t-1}\right|>0;\\ \begin{array}{c} v_{ij}\left(t\right)=\sum_{k=1}^{M_{1}}\alpha_{k}x_{i_{k}j}\left(t-1\right)-\sum_{k=1}^{M_{\text{E}}}\beta_{k}x_{i_{k}j}\left(t-1\right)\\ \text{Rand}\left(0,1\right)\ldots 0.5,\;\left|C_{R}^{t-1}\right|>0; \end{array} \end{array}\\ v_{ij}\left(t\right)=x_{ij}\left(t-1\right),\;{\text{IaIaS}}_{i}\left(t\right)={\text{IaIaS}}_{i}\left(t-1\right),\\ \left|C_{R}^{t-1}\right|=0. \end{array} \end{array}\right. \end{array}$$
Equation (11) not only integrates the characteristics of the S-S operator and the S-R operator but also avoids the similarity with the S-R operator.
(9) R-R operator. Let the characteristics *j* of the *L* individuals in the set *C*_PR_^*t*−1^ and their average state values pass to the corresponding features *j* of the cured and obtained immune individuals *i* to enhance their constitution, that is,
  (12) $$\begin{array}{c} \left\{\begin{array}{c} v_{ij}\left(t\right)=\frac{1}{\left|C_{\text{PR}}^{t-1}\right|}\sum_{k\in C_{\text{PR}}^{t-1}}x_{kj}\left(t-1\right),\;\left|C_{\text{PR}}^{t-1}\right|>0;\\ v_{ij}\left(t\right)=x_{ij}\left(t-1\right),\;{\text{IaIaS}}_{i}\left(t\right)={\text{IaIaS}}_{i}\left(t-1\right),\\ \left|C_{\text{PR}}^{t-1}\right|=0. \end{array}\right. \end{array}$$
(10) *R-S (ω) Operator*. Let the feature *j* of the *L* individuals in the set *C*_*s*_^*t*−1^ and their average state values be transmitted to the corresponding features *j* of the cured and immunized individual *i*, respectively, so that the immunity disappears, that is,
  (13) $$\begin{array}{c} \left\{\begin{array}{c} v_{ij}\left(t\right)=\frac{1}{\left|C_{S}^{t-1}\right|}\sum_{k\in C_{S}^{t-1}}x_{kj}\left(t-1\right),\;\left|C_{S}^{t-1}\right|>0;\\ v_{ij}\left(t\right)=x_{ij}\left(t-1\right),\;{\text{IaIaS}}_{i}\left(t\right)={\text{IaIaS}}_{i}\left(t-1\right),\\ \left|C_{S}^{t-1}\right|=0. \end{array}\right. \end{array}$$
Although the state transition of R⟶S needs to be delayed by *ω* periods, the state value of the feature of the period *t* individual *i* being attacked by the virus is only related to the state value of the period *t* − 1, for the same reason as the *E*– *I*(*ω*) operator.
(11) *Growth Operator*. A new generation of individuals is compared with the corresponding previous generation individuals, and the better ones are updated into the next generation of individuals, and the poorer ones remain unchanged until they are changed. For the minimization optimization problem formula (13), the growth operator can be described as
  (14) $$\begin{array}{c} X_{i}\left(t\right)\left\{\begin{array}{c} V_{i}\left(t\right),\;\text{IPI}\left(V_{i}\left(t\right)\right)>\text{IPI}\left(X_{i}\left(t\right)\right);\\ X_{i}\left(t-1\right),\;\text{PI}\left(V_{i}\left(t\right)\right)⩽\text{IPI}\left(X_{i}\left(t\right)\right); \end{array}\right.\;i=1,2,\ldots ,N. \end{array}$$

In equation (14), the functions IPI(*V*_*i*_(*t*)) and IPI(*X*_*i*_(*t*)) are calculated according to equation (12).

The operators are constructed according to the characteristics of the IAIA infectious disease model, which are unique operators describing the running process of the model. Therefore, these operators are only related to the IAIA infectious disease model, which was first proposed internationally. If the infectious disease model is different, the construction method of the relevant operator will be different. The IAIA algorithm utilizes S-S, S-E, S-R, E-E, *E*– I(*ω*), E-R, I-I, I-R, R-R, and R-S(*ω*) operators to exchange information between individuals. Individuals with a high *IPI* index pass strong E-E, I-I, R-R, and other operators to transmit strong feature information to individuals with low *IPI* index, so that individuals with low *IPI* index can develop in a good direction. S-E, S-R, E-I(*ω*), and R-S (*ω*) operators enable individuals in different states to obtain average feature information of other individuals, thereby reducing the probability that individuals fall into local optimum; The S-S operator can increase the activity of the individual, thus expanding the search range; the E-R and I-R operators have both the characteristics of the S-S operator and the characteristics of the S-E, S-R, E-I (*ω*), and R-S(*ω*) operators.

It can be known from the IAIA algorithm that the ecosystem is a discrete space, but the period *t* of each individual *X*_*i*_(*t*)(*i*=1,2,…, *N*) is the value of the continuous real space. The total number of individuals is *N*, and each individual is a trial solution of the optimization problem formula (1). The objective function value is *F*(*X*_*i*_(*t*)) (calculated according to equation (2)), then the set of states of all individuals is

(15) $$\begin{array}{c} \left\{\begin{array}{c} F_{\text{state}}=\left\{F_{1},F_{2},\ldots ,F_{N}\right\},\;F_{1}<F_{2}<\ldots <F_{N};\\ F_{\text{state}}=\left\{F\left(X_{i}\left(t\right)\right)|X_{i}\left(t\right)\in H\right\}. \end{array}\right. \end{array}$$

Without loss of generality, let *F*_1_ be the global optimal solution we seek. The subscripts of equation (15) are taken out to form a set, is *U*={1,2,…, *N*}.

The elements in the set *U* are the states in which each individual may be in a random search. Suppose that the best target function value we searched for at a certain time is *F*_*i*_ and its corresponding state is *i*. Obviously, it is known from equation (15) that if searching for a better state *k* during the next period of search, then *k* < *i* should be satisfied; conversely, if it is shifted to a worse state *k*, then *k*  >  *i* should be satisfied.

 
∀**X** ∈ *H*, *F*_1_⩽*F*(**X**)⩽*F*_*N*_. If a state with the same objective function value is merged into a set, then *H* can be divided into nonempty subsets as:

(16) $$\begin{array}{c} \left\{\begin{array}{c} X_{S}^{i}=\left\{X|X\in H,F\left(X\right)=F_{i}\right\},\;i=1,2,...,N;\\ \overset{N}{\underset{i=1}{\sum }}\left|X_{S}^{i}\right|=N;\;\forall i\in \left\{1,2,\ldots ,N\right\},\;X_{S}^{i}\neq \phi ;\\ \forall i\neq j,\;X_{S}^{i}\cap X_{S}^{j}=\phi ,\;\overset{N}{\underset{i=1}{\cup }}X_{S}^{i}=H. \end{array}\right. \end{array}$$

Obviously, state switching in *X*_*s*_^*i*^ does not change the value of the objective function.

Let **X**^*i*,*j*^(*i*=1,2,…, *N*, *j*=1,2,…, |*X*_*S*_^*i*^|) denote the *j* state in *X*_*S*_^*i*^. During the evolution of an individual, the transition from one state (*i*, *j*) to another state (*k*, *l*) can be expressed as *X*^*i*,*j*^ ⟶ *X*^*k*,*l*^, which assumes: From *X*^*i*,*j*^ to *X*^*k*,*l*^ the transition probability of an individual is *p*_*ij*,*kl*_ and the transition probability from any state of *X*^*i*,*j*^ to *X*^*k*,*s*^ is *p*_*ij*,*k*_, from any state of *X*_*s*_^*i*^ to *X*^*k*,*s*^, the transition probability of the state is *p*_*i*,*k*_, that is:

(17) $$\begin{array}{c} \left\{\begin{array}{c} p_{i,k}\geq p_{i,j,k}⟶\sum_{k=1}^{N}p_{i,k}\geq \sum_{k=1}^{N}p_{ij,k}=1;\\ 0\leq \sum_{k=1}^{N}p_{i,k}\leq 1,\\ \sum_{k=1}^{N}p_{i,k}=1. \end{array}\right. \end{array}$$

From those steps, we can establish an algorithm (Algorithm 1).

## 4. Experimental Analysis

The experiments were conducted using real data and simulated scenes. The experimental design is as follows:

1. The population of Pterospermum heterophyllum is 215,668 and is distributed in the southwestern part of Hunan Province with an area of 287,640 m^2.^
2. Simulation using OMNET++ platform was used
3. Comparison algorithm: the status estimation and cache management algorithm (SECM) [26], the information cache management and data transmission algorithm (ICMT) [27]
4. Parameter settings: all the parameter settings in this study are shown in Table 4

## 5. Results and Discussion

**Figure 3:**
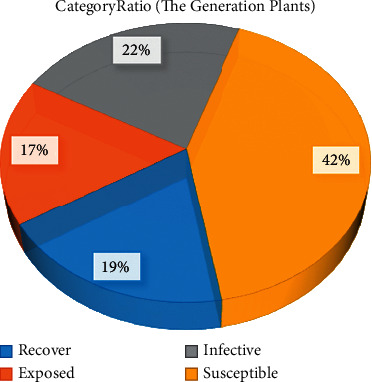
Classification of various types of plants in the initial state.

Figure 3 shows the classification of various types of plants in the initial state. Among these plants, the susceptible, exposed, infective, and recover classes accounted for 42%, 17%, 22%, and 19% of the total population, respectively. It should be noted that in the initial state of the plants, the susceptible class accounts for almost half. If this class is transformed into the infective class in large numbers, it will be unfavorable for the spread of this plant.

**Figure 4:**
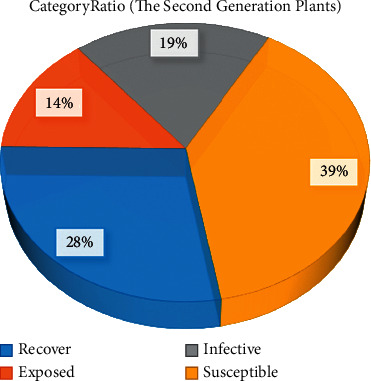
Classification of second-generation plants after using the IAIA algorithm.

Figure 4 shows the classification of the second generation plants after using the IAIA algorithm. The proportions of susceptible class, exposed class, and infective class declined, and each of these three classes has fallen by 3%. Among them, although the proportion of the susceptible class is still the largest, reaching 39%, the recovery class, which is most conducive to growth and development, has increased from 19% to 28%. This finding indicates that disease was further improved by superior seed selection, seed isolation methods, and plant labeling.

**Figure 5:**
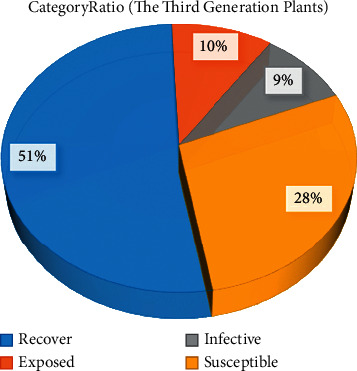
Classification of three generations of plants after using the IAIA algorithm.

**Figure 6:**
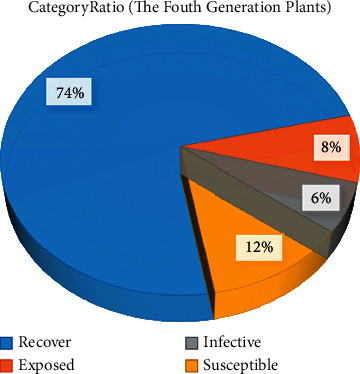
Classification of four generations of plants after using the IAIA algorithm.

Figures 5 and 6 show the classification of the third and fourth generation plants after using the IAIA algorithm, respectively. Compared with the other groups, more plants were labeled under the recover class by the choice of superior partners, that is, they have increased from the initial 19% to 51% in the third-generation plant classification, and by the fourth-generation plant classification, they have reached 74%. The susceptible class that initially accounted for the largest proportion continued to decline as the dominant plants were selected. In the third generation of plant classification, its proportion dropped by half. In the fourth generation of plant classification, the susceptible class was only 12%, accounting for about a quarter of the original. And exposed and infective classes, which are relatively unfavorable for plant growth and development, dropped from 17% and 22% to 8% and 6%, respectively. These indicate that more high-quality genes have been retained through the use of the proposed algorithm.

**Figure 7:**
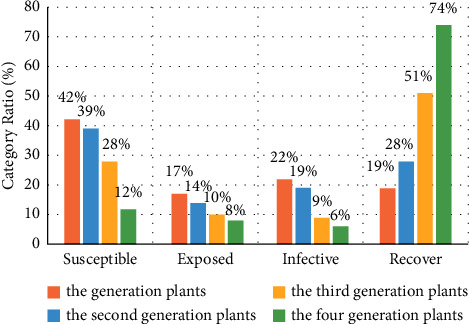
Changes in the proportion of each class in different generations after using the IAIA algorithm.

Figure 7 shows the overall change trend of the proportions of the four classifications of plants. The four bars of each class represent the proportion of the initial, second, third, and fourth generations from left to right. According to the figure, we can know that by using the IAIA algorithm, the proportion of recover class that carries high-quality genes continues to increase rapidly, from 19% in the initial state to 74% in the fourth generation of plant classification, an increase of approximately 3 times. In the fourth generation of plant classification, the total proportions of susceptible, exposed, and infectious species accounted for only 26%. It shows that after the IAIA algorithm is used to select dominant plants, the number of plants with disease genes is greatly reduced.

As determined through the IAIA algorithm, the cooperation and classification of plants after absorbing immune seeds can significantly eliminate the effects of plant-borne diseases and show desirable effects on the cultivation of rare plants. Through simulation of the algorithm, this advantageous selection method can be applied to actual cultivation.

To verify the success rate of the algorithm for the recovery class, we used SECM and ICMT for comparison with the IAIA algorithm.

**Figure 8:**
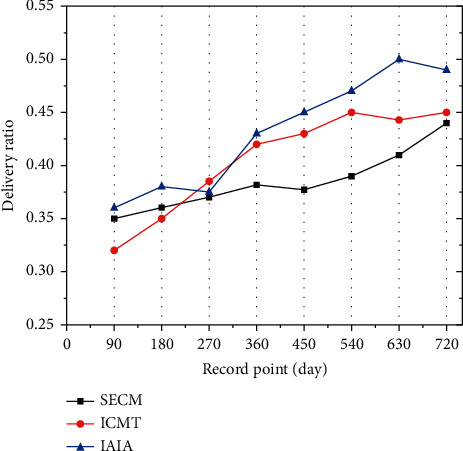
Generation delivery ratio of the recovery class.

Figure 8 shows the delivery ratio of plant generation in the recovery class. The figure shows the similar transmission delivery ratio of the three algorithms for the recover class seed. By the 270th day, the IAIA algorithm delivery ratio reached 37.5%, which is between the other two algorithms. However, by the 720th day, the IAIA algorithm delivery ratio reached 49%, and that of the other two algorithms totaled 45% and 44%. By natural selection, no singular method exceeded a 50% transmission delivery ratio. It shows that with the increase of time, the delivery rate of the IAIA algorithm proposed in this paper is the most stable in plant generation, which is more conducive to the selection of dominant plants.

**Figure 9:**
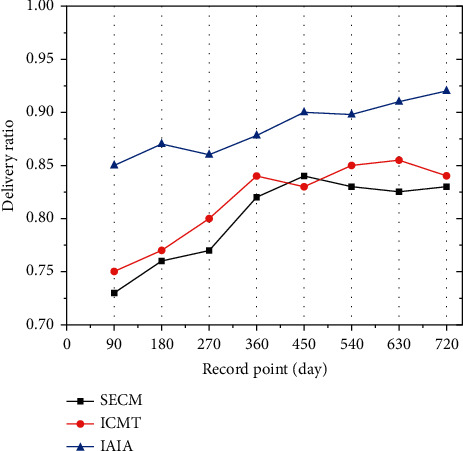
Transmission delivery ratio of second-generation plants in the recovery class.

Figure 9 shows the transmission delivery ratio of second-generation plants in the recover class. Through the classification mark of the first recovery class, the transmission delivery ratio of IAIA algorithm recovery class exceeded 85%. This result was due to the large number of plants that have achieved an increased acceptance rate for seeds labeled with immune signals, resulting in the easy use of the recovery class in the selection process. On the other hand, the transmission delivery ratio of SECM and ICMT recovery class is less than 85%. Because they are not as efficient as IAIA in seed recognition, it leads to a decrease in the acceptance rate of immune seeds and is not conducive to the immune effect of plants against epidemics.

**Figure 10:**
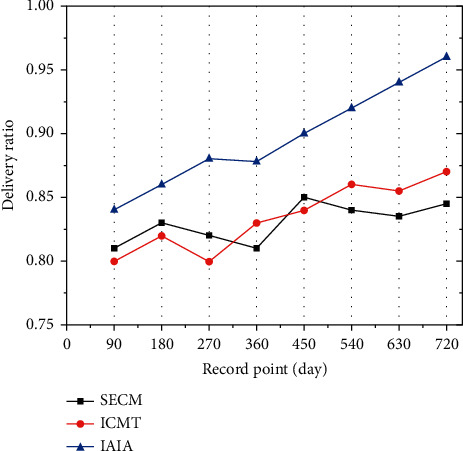
The transmission delivery ratio of third-generation plants in the recover class.

Figure 10 shows the delivery ratio of the third-generation plants in the recover class. With the third mark, the IAIA algorithm recovery class showed a transmission delivery ratio of over 90%. On the contrary, the recovery class transmission rate of the other two algorithms is less than 90%. By guiding the plants to receive the seeds of the labeled recovery class, the plants can selectively resist the epidemic transmission of the virus, ultimately achieving immunity in a large area. Due to the slow transmission efficiency of SECM and ICMT, plants could not receive recovery seeds in time, resulting in a slow increase in the number of immune plants, and death of some plants due to infectious diseases.

Three sets of experiments are used to simulate the selection of plant seeds in opportunistic social networks. Among them, the effective marking and adding of the recover class seeds are similar to the key data of prioritization. It can be selected by a large number of plants, thereby reducing the epidemiology of rare plants. In actual plant reproduction, the use of DNA markers to preferentially mark the recover class seeds can effectively improve plant immunity and realize the selection of dominant plants.

## 6. Conclusion

In this research, we selected the rare and endangered Pterospermum heterophyllum Dutch plant as the research object and applied it to an opportunistic social network. All plants were considered as nodes and all seeds as transmitted data. Through the use of historical records, the receipt of seeds was recorded to establish infection and immunity algorithms in opportunistic social networks. Experimental results show that our proposed method can effectively identify healthy plants and reduce diseased plants. It realizes the selectivity of dominant plants in the social opportunity network.

In future works, we will use genetic markers to mark each type of seed and record the path and coverage of high-quality seed propagation over prolonged periods to provide a better way of studying disease immunity.

## Figures and Tables

**Algorithm 1:**
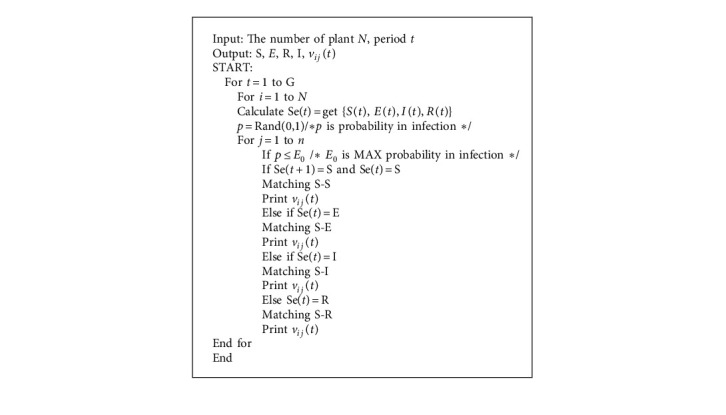
Infection and immunity algorithm in opportunistic social networks (IAIA).

**Table 1:** Summary of related studies.

References	Method used	Research significance
Literature [7]	Genetic algorithm	Classification of plant diseases
Literature [8]	RF, SVM, DT, KNN, and Naive Bayes	
Literature [9]	PCA-whale optimization algorithm, DNN	
Literature [10]	Create background database, mathematical algorithms, and decision models	Identification of weeds and crops
Literature [11]	Mathematical derivation	Plant growth mechanism planning
Literature [15]	Bio-inspired computing algorithm	
Literature [12]	Crow search algorithm, DNN	Improved plant genetic quality
Literature [13]	Plant gene regulation algorithm	
Literature [14]	Machine learning algorithms, multilayer perceptron, SVM, RF	

**Table 2:** Some symbols and their explanations.

Symbol	Meaning
*N*(*t*)	Total number of individuals during period *t*
*S*(*t*)	Number of susceptible individuals during period *t*
*E*(*t*)	Number of exposed individuals during period *t*
*I*(*t*)	Number of infected individuals during period *t*
*R*(*t*)	Number of recovered individuals during period *t*
*ω*_1_	Latent cycle
*ω*_2_	Immune cycle
*L*	Number of randomly selected individuals
*p*	State transition probability
**V**_*i*_(*t*)	The characteristics set of the period *t*
IPI	The individual's physical strength
*U*	The subscript set of the global optimal solution
*M*_*I*_, *M*_*E*_	The number of active individuals involved in information exchange
IaIaS_*i*_(*t*)	The state of the individual *i* at the period *t*, which is one of the four states *S*, *E*, *I*, and *R*.
*F*_state_	The set of states of all individuals

**Table 3:** Taking value method of parameters in the IAIA model.

Name of parameter	Meaning of parameter	Method of taking value
*β* ^*t*^	Effective contact rate of the disease	*β* ^*t*^=Rand(0,1)
*α* ^*t*^	The proportion of individuals transferring from the latent state into the disease state	*α* ^*t*^=Rand(0,1)
*γ* ^*t*^	The proportion of infected individuals transferred into cured individuals	*γ* ^*t*^=Rand(0,1)
*b* ^*t*^	Direct immunization rate of individuals	*b* ^*t*^=Rand(0,1)
*u* ^*t*^	The proportion of individuals transferring from the latent state into the immune state	*u* ^*t*^=Rand(0,1)

**Table 4:** Experimental parameters and their values.

Parameter	Value
Simulation time	720 days
Simulation area	287,640 m^2^
Number of plants in the simulation node	215,668
Seed release cycle	146–182 days
Seed release method	Random
Maximum survival time of seeds	4 days
Seed movement speed	3.5 km/h
Plant receiving the maximum number of seeds	50/time

## Data Availability

Data used to support the findings of this study are currently under embargo while the research findings are commercialized. Requests for data, 12 months after publication of this article, will be considered by the corresponding author.
